# Association of Modifiable Lifestyle and Metabolic Factors With the Risk of Developing Sepsis: 2-Sample Mendelian Randomized Study

**DOI:** 10.2196/72244

**Published:** 2025-11-03

**Authors:** Haifeng Lv, Jing Liu, Yelin Cao, Weina Fan, Guojie Shen, Feifei Wang, Qingqing Ye, Xiaoliang Wu, Kaijin Xu

**Affiliations:** 1 Department of Intensive Care Unit, The First Affiliated Hospital, Zhejiang University School of Medicine, Hangzhou, China; 2 Department of Hepatology, The Affiliated Hospital of Hangzhou Normal University, Hangzhou, China; 3 State Key Laboratory for Diagnosis and Treatment of Infectious Diseases, National Clinical Research Center for Infectious Diseases, China-Singapore Belt and Road Joint Laboratory on Infection Research and Drug Development, National Medical Center for Infectious Diseases, Collaborative Innovation Center for Diagnosis and Treatment of Infectious Diseases, The First Affiliated Hospital, Zhejiang University School of Medicine, No.79 Qingchun Road, Shangcheng District, Zhejiang Province, Hangzhou, 310003, China, 86 13750870030; 4 Yuhang Institute for Collaborative Innovation and Translational Research in Life Sciences and Technology, Hangzhou, China

**Keywords:** Mendelian randomization, sepsis, modifiable factors, lifestyle, metabolism

## Abstract

**Background:**

Sepsis is a life-threatening condition characterized by organ dysfunction resulting from dysregulated host response to infections. Approximately 48.9 million people worldwide are diagnosed with sepsis annually, leading to 11 million deaths and representing 19.7% of all global deaths. No specific, effective treatments for sepsis, which has a poor prognosis, are available.

**Objective:**

The study aimed to systematically explore the association between genetically predicted modifiable risk factors and sepsis.

**Methods:**

Univariable 2-sample Mendelian randomization (MR) analysis was performed to explore the association between 30 modifiable risk factors (12 lifestyle, 3 educational and psychological, and 15 metabolic factors) and sepsis. Heterogeneity was evaluated using the Cochran *Q* analysis. Sensitivity analyses were conducted using the MR-Egger regression intercept tests and leave-one-out analyses. Additionally, multivariable MR analyses were performed to adjust for genetic associations between the instruments and obesity.

**Results:**

Genetically predicted smoking (odds ratio [OR] 1.20, 95% CI 1.06‐1.36; *P*=.005), a higher number of cigarettes smoked daily (OR 1.70, 95% CI 1.29‐2.23; *P*<.001), a higher overall health rating (OR 2.19, 95% CI 1.61‐2.98; *P*<.001), BMI (OR 1.50, 95% CI 1.38‐1.63; *P*<.001), waist circumference (OR 1.70, 95% CI 1.53‐1.89; *P*<.001), whole body fat mass (OR 1.50, 95% CI 1.37‐1.64; *P*<.001), trunk fat mass (OR 1.48, 95% CI 1.36‐1.62; *P*<.001), arm fat mass (OR 1.57, 95% CI 1.43‐1.71; *P*<.001), and leg fat mass (OR 1.69, 95% CI 1.51‐1.90; *P*<.001) were associated with increased sepsis risk. However, light physical activity (OR 0.26, 95% CI 0.08‐0.83; *P*=.03), higher education attainment (OR 0.52, 95% CI 0.40‐0.67; *P*<.001), and high-density lipoprotein cholesterol (OR 0.91, 95% CI 0.84‐0.98; *P*=.02) exhibited protective effects against sepsis. Using a multivariate analysis of obesity traits, the waist circumference (OR 2.16, 95% CI 1.18‐3.96; *P*=.01) was an independent risk factor of sepsis.

**Conclusions:**

Our study demonstrated that genetic predictors of lifestyle (smoking and physical activity), educational level, and metabolic factors (waist circumference and high-density lipoprotein cholesterol) exhibited a causal association with sepsis risk. Future research should further investigate the underlying mechanisms of these associations to inform more effective preventive strategies against sepsis.

## Introduction

Sepsis is a life-threatening condition characterized by organ dysfunction resulting from a dysregulated host response to infections [1]. Epidemiological studies indicate that approximately 48.9 million people worldwide were diagnosed with sepsis annually, leading to 11 million deaths and representing 19.7% of all global deaths [2]. In China, sepsis affects 20% of patients in intensive care units, with a 90-day mortality rate of 35.5% [3]. The substantial epidemiological burden underscores the critical importance of sepsis as a public health issue [4]. Despite decades of research, current therapies remain limited to organ support and antimicrobial interventions, without any targeted treatment available [5-7]. These limited therapeutic options highlight the urgent need for shifting focus toward modifiable risk factors for prevention. The World Health Organization 2020 Sepsis Report specifically highlights vulnerable populations and health care seekers, while outlining future research directions and priorities in sepsis epidemiology. Although sepsis manifests diversely and can be life-threatening, it remains preventable and reversible [4].

Host risk factors for sepsis are multifaceted, encompassing extremes of age, immunosuppression, and comorbidities [8,9]. Emerging evidence suggests that socioeconomic determinants (eg, education level and occupation) and metabolic disorders (eg, obesity and diabetes) may influence susceptibility to sepsis [10-16]. However, the existing observational studies report inconsistent conclusions. For instance, although lower socioeconomic status is broadly linked to higher sepsis incidence [10,11], another study paradoxically found no association using adjusted models [17]. Metabolic comorbidities further illustrate this ambiguity. Although obesity is widely implicated in sepsis risk [18], some observational studies found that individuals with obesity showed a lower incidence and fatality than did those with underweight among patients with sepsis. These discrepancies may stem from methodological limitations in observational research, such as residual confounding and reverse causality. For instance, sepsis-induced systemic inflammation could transiently alter metabolic markers, obscuring true causal directions.

The core idea of Mendelian randomization (MR) is to use genetic variants (typically single-nucleotide polymorphisms [SNPs]) as instrumental variables to infer whether a potential causal association exists between an exposure and a health outcome. Recent MR studies have explored individual factors such as obesity, diabetes mellitus, and lipid profiles in patients with sepsis [19-23]; however, critical gaps persist. First, lifestyle factors (eg, smoking and diet) remain understudied despite their clinical relevance. Second, the existing MR analyses of sepsis predominantly examine isolated risk factors, neglecting potential synergies between socioeconomic, metabolic, and behavioral determinants [8,9,24-28]. Third, the interaction between modifiable factors and genetic predisposition remains unexplored, limiting personalized preventive strategies.

To address these gaps, we conducted a comprehensive 2-sample MR analysis of 30 modifiable factors spanning lifestyle, socioeconomic status, and metabolic health. This approach not only circumvents confounding biases but also systematically evaluates multifactorial contributions to sepsis pathogenesis. Our findings aimed to clarify controversial associations in prior literature and prioritize actionable targets for intervention.

## Methods

### MR Design

**Figure 1.**
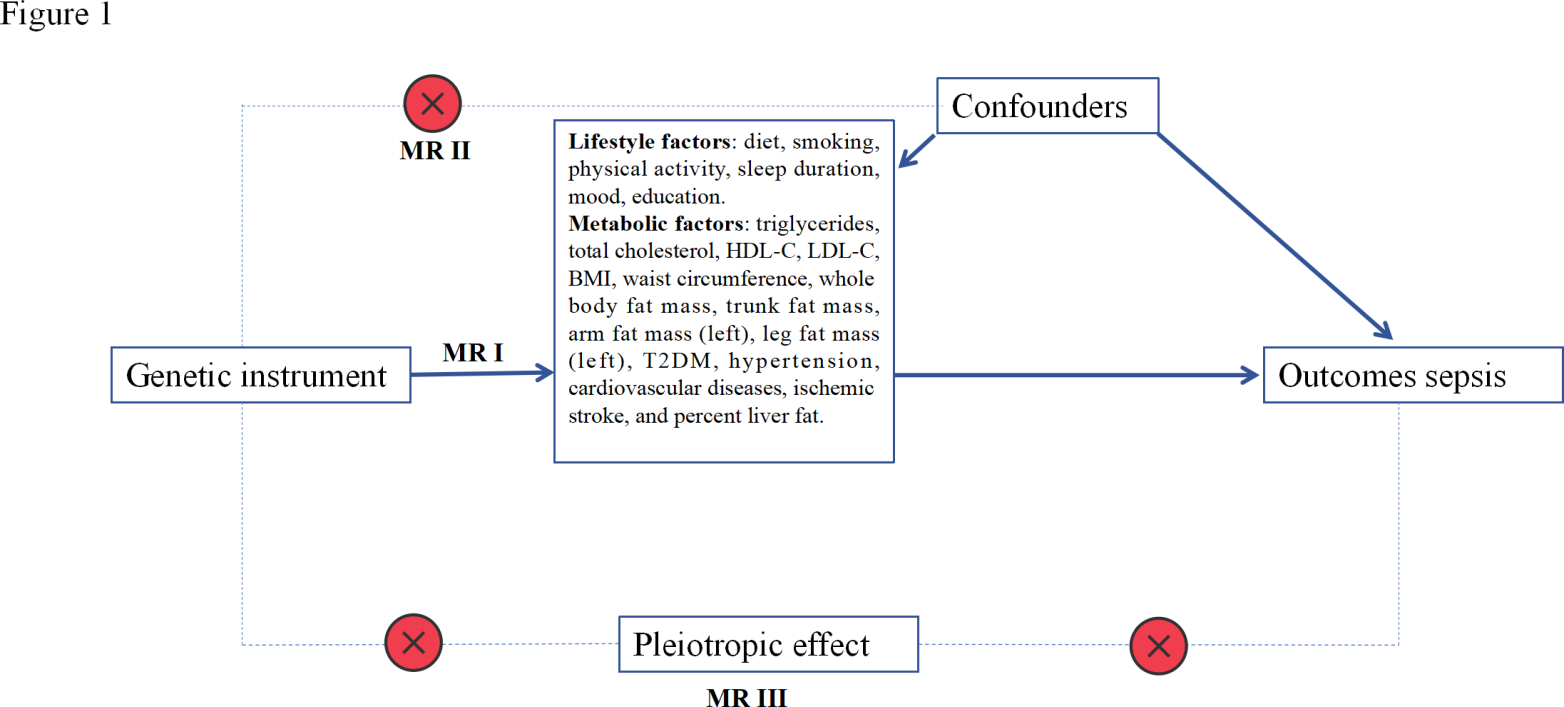
Overview of the design and methods used in this Mendelian randomization (MR) study. MR analysis was used to explore the causal relationships, including the following groups: lifestyles and metabolic factors. Solid arrows represent causal effects, and dashed arrows and crosses represent causal effects prohibited by MR assumptions II and III. HDL-C: high-density lipoprotein cholesterol; LDL-C: low-density lipoprotein cholesterol; T2DM: type 2 diabetes mellitus.

MR is based on three key assumptions: genetic variants are (1) linked to risk factors, (2) independent of confounding factors, and (3) influence outcomes solely through risk factors (Figure 1). A total of 30 prominent modifiable risk factors were included and grouped into lifestyle and metabolic factors. This study adhered to the Strengthening the Reporting of Observational Studies in Epidemiology using Mendelian Randomization (STROBE-MR) guidelines. Initially, we screened instrumental variables for MR analysis using lifestyle factors and metabolic comorbidities as “exposures” and sepsis as “outcomes.” Subsequently, we conducted a reverse MR analysis, with sepsis as the “exposure” and lifestyle and metabolic factors as the “outcomes.”

### Data Sources

Genome-wide association study (GWAS) data of sepsis were obtained from the OpenGWAS database, a dataset published by the UK Biobank in 2021, comprising a sample of 486,484 participants from European populations. Additionally, we used 30 parameters from the Integrative Epidemiology Unit OpenGWAS Project as our lifestyle, education and psychology, and metabolic traits. Data are listed in Table 1.

**Table 1.** Characteristics of the genome‐wide association study (GWAS) summary data in this study.

Exposure	Ethnicity	GWAS ID	Total population, n	SNPs^a^, n
Diet				
Alcohol consumption	European	ieu-b-73	335,394	11,887,865
Coffee consumption	European	ukb-b-5237	428,860	9,851,867
Milk consumption	South Asian	ukb-e-100520_CSA	1469	9,797,409
Sweets consumption	African American	ukb-e-102330_AFR	1207	15,533,528
Smoking				
Smoking initiation	European	ieu-b-4877	607,291	11,802,365
Age of smoking	European	ieu-b-24	341,427	11,894,779
Number of cigarettes daily	European	ukb-b-6019	108,946	9,851,867
Physical activity				
Light activit**y**	European	ukb-b-11495	460,376	9,851,867
Moderate to vigorous	European	ebi-a-GCST006097	377,234	11,808,007
Sleep habit				
Insomnia	European	ukb-b-3957	462,341	9,851,867
Nap during day	European	ukb-b-4616	462,400	9,851,867
Sleep duration	European	ukb-b-4424	460,099	9,851,867
Physical and mental				
Unhealthy emotions	European	ukb-b-6991	459,560	9,851,867
Overall health rating	European	ukb-b-16489	458,079	9,851,867
College or university	European	ukb-b-6306	460,844	9,851,867
Blood lipid traits				
Triglycerides	European	ieu-b-4850	78,700	7,892,037
Total cholesterol	European	met-d-Total_C	115,078	12,321,875
HDL‐c^b^	European	met-d-HDL_C	115,078	12,321,875
LDL‐c^c^	European	ie,u-b-110	440,546	12,321,875
Obesity traits				
BMI	European	ukb-b-19953	461,460	9,851,867
Waist circumference	European	ukb-b-9405	462,166	9,851,867
Whole body fat mass	European	ukb-b-19393	454,137	9,851,867
Trunk fat mass	European	ukb-b-20044	454,588	9,851,867
Arm fat mass (left)	European	ukb-b-8338	454,684	9,851,867
Leg fat mass (left)	European	ukb-b-7212	454,823	9,851,867
Metabolic syndrome				
T2DM^d^	European	ebi-a-GCST010118	433,540	11,222,507
Hypertension	European	finn-b-I9_HYPTENS	162,837	16,380,466
Cardiovascular diseases	European	finn-b-I9_CVD	107,684	16,380,466
Ischemic stroke	European	ebi-a-GCST006908	440,328	8,296,492
Percent liver fat	European	ebi-a-GCST90016673	32,858	9,275,407

a SNP: single-nucleotide polymorphism.

b HDL‐C: high-density lipoprotein cholesterol.

c LDL‐C: low-density lipoprotein cholesterol.

d T2DM: type 2 diabetes mellitus.

### Selection of Genetic Variants

In this MR analysis, instrumental variables were used to investigate the association between potentially modifiable risk factors and sepsis. These risk traits were categorized into three main parts: (1) lifestyle factors encompassing 4 dietary traits, 3 smoking-related traits, 2 pertaining to physical activity, and 3 regarding sleep habits; (2) educational and psychological factors, including physical and mental well-being and education level; and (3) metabolic factors comprising 4 traits linked to blood lipid parameters, 6 associated with obesity traits, and 5 related to metabolic comorbidities. SNPs were selected as independent genetic predictors based on the following criteria: first, a genome-wide significance threshold of *P*<5×10^−8^ was applied. If there were insufficient significant SNPs under this threshold, a threshold of *P*<1×10^−5^ was used. Second, the clump function was used to test for linkage disequilibrium with a threshold of *R*^2^<0.001 and a distance of 10,000 kb. Third, SNPs related to outcomes were excluded using the linkage disequilibrium link database to avoid confounding factors. Fourth, SNPs with an *F* statistic <10 were excluded to avoid bias from weak instruments. The proportion of exposure variance explained by genetic instruments (*R*^2^) was calculated to quantify the strength of the genetic tools. The *F* statistic for each SNP was calculated to assess the strength of the selected instruments.

### Ethical Considerations

This study is a secondary analysis of publicly available GWAS summary statistics obtained from the Integrative Epidemiology Unit OpenGWAS database [29] as shown in Table 1. These datasets consist of deidentified data from participant studies approved by an ethics committee concerning human experimentation, comply with the ethical principles of the Declaration of Helsinki, and were approved by the Ethics Committee of the First Affiliated Hospital of Zhejiang University (2025B-1097).

### Statistical Analysis

In this study, all analyses were performed using the *TwoSampleMR* package in R (version 4.2.1; R Foundation for Statistical Computing). The random-effects inverse variance weighted (IVW) method was used as the primary outcome; this method assumed that all instrumental variables (SNPs) were valid instruments (ie, satisfying the exclusion restriction assumption) and estimated the causal effect between exposure and outcome by calculating the weighted regression slope of the SNP-outcome associations on the SNP-exposure associations. Although the IVW approach offered the highest statistical power among MR methods, it was sensitive to directional pleiotropy, which might have biased causal estimates if invalid SNPs were present. Therefore, the MR-Egger and weighted median methods were used to refine the IVW estimation. These alternative methods offered more dependable estimates across a broader spectrum of scenarios, albeit with a trade-off of reduced efficiency, resulting in wider CIs. MR-Egger permitted all genetic variants to exhibit pleiotropy; however, pleiotropy must be independent of variant exposure associations. Meanwhile, the weighted median method allowed the incorporation of potentially invalid instrumental variables under the assumption that at least half of the instruments used in the MR analysis were valid. We performed reverse MR analyses by swapping the roles of the original exposure and outcome variables; IVW analysis tests were conducted using the same set of SNPs.

Heterogeneity was evaluated using the Cochran *Q* analysis. Sensitivity analyses were conducted using MR-Egger regression intercept tests and leave-one-out analyses, with statistical significance set at *P*<.05. Additionally, multivariable MR analyses were performed to adjust for the genetic association of the instruments with the BMI.

## Results

### Baseline Characteristics

We assessed 30 potentially modifiable risk factors to investigate their causal associations with sepsis and categorized them into 3 groups (Table 1): lifestyle, educational and psychological, and metabolic factors. The number of SNPs ranged between 7,892,037 and 15,533,528 (Table 1). Notably, the *F* statistics for all considered traits exceeded 10, indicating the absence of a potential weak instrumental bias.

### Lifestyle Factors

Regarding the investigation of lifestyle factors, our findings revealed that genetically predicted smoking initiation (odds ratio [OR] 1.20, 95% CI 1.06‐1.36; *P*=.005) and a higher number of cigarettes smoked daily (OR 1.70, 95% CI 1.29‐2.23; *P*<.001) were associated with an increased risk of sepsis. Conversely, genetically predicted light physical activity (OR 0.26, 95% CI 0.08‐0.83; *P*=.02) was associated with a decreased risk of sepsis. These results remained consistent across the random-effects IVW and weighted median analyses (Table 2). Notably, no significant causal association was detected between genetically predicted alcohol, coffee, milk, and sweet consumptions; age at smoking; moderate to vigorous physical activity; insomnia; daytime napping; sleep duration; and sepsis (all *P*>.05). We did not find statistically significant results when investigating reverse causality validation (*P*>.05).

**Table 2.** Mendelian randomization (MR) estimates for the causal effect of modifiable lifestyle and metabolic factors on sepsis.

Exposure	SNPs^a^, n	IVW^b^, OR^c^ (95% CI)	*P* value	WM^d^, OR (95% CI)	*P* value	MR-Egger, OR (95% CI)	*P* value	Cochran *Q*–derived *P* value	MR-Egger intercept–derived *P* value
Diet									
Alcohol consumption	35	1.00 (0.76‐1.32)	.99	1.04 (0.69‐1.57)	.85	1.02 (0.64‐1.61)	.94	.34	.92
Coffee consumption	39	1.14 (0.79‐1.64)	.48	1.10 (0.71‐1.71)	.67	0.88 (0.42‐1.83)	.73	.06	.43
Milk consumption	5	0.97 (0.92‐1.03)	.29	0.95 (0.89‐1.02)	.19	1.29 (0.73‐2.28)	.45	.71	.40
Sweets consumption	11	0.99 (0.97‐1.01)	.47	0.99 (0.97‐1.02)	.64	1.05 (0.99‐1.11)	.110	.25	.06
Smoking									
Smoking initiation	86	1.20 (1.06‐1.36)	.005	1.22 (1.02‐1.47)	.034	1.72 (0.90‐3.28)	.10	.41	.27
Age of smoking	7	0.89 (0.46‐1.71)	.723	1.25 (0.56‐2.79)	.59	0.32 (0.04‐2.68)	.34	.28	.37
Number of cigarettes daily	6	1.70 (1.29‐2.23)	<.001	1.72 (1.25‐2.36)	<.001	2.30 (1.31‐4.04)	.04	.86	.29
Physical activity									
Light activity	12	0.26 (0.08‐0.83)	.03	0.12 (0.03‐0.58)	.007	1.12 (0.02‐78.84)	.96	.74	.50
Moderate to vigorous	19	0.98 (0.61‐1.57)	.92	1.10 (0.58‐2.06)	.78	0.27 (0.03‐2.58)	.27	.68	.27
Sleep habit									
Insomnia	38	1.08 (0.63‐1.87)	.77	1.02 (0.50‐2.07)	.95	0.52 (0.09‐2.94)	.46	.02	.39
Nap during day	88	1.04 (0.74‐1.45)	.84	0.93 (0.55‐1.57)	.79	1.45 (0.46‐4.55)	.53	.45	.55
Sleep duration	65	0.80 (0.57‐1.11)	.19	0.83 (0.50‐1.36)	.45	1.17 (0.32‐4.32)	.81	.27	.55
Physical and mental									
Unhealthy emotions	41	1.36 (0.67‐2.74)	.40	1.22 (0.45‐3.30)	.70	0.48 (0.01‐22.66)	.71	.81	.59
Overall health rating	106	2.19 (1.61‐2.98)	<.001	1.63 (1.09‐2.44)	.02	2.33 (0.49‐11.00)	.29	.02	.94
College or university	242	0.52 (0.40‐0.67)	<.001	0.55 (0.37‐0.82)	.003	0.52 (0.18‐1.51)	.23	.85	.97
Blood lipid traits									
Triglycerides	38	1.00 (0.89‐1.13)	.94	1.02 (0.88‐1.18)	.80	1.14 (0.91‐1.42)	.25	.008	.20
Total cholesterol	359	0.97 (0.89‐1.05)	.47	0.98 (0.87‐1.10)	.70	1.00 (0.88‐1.14)	.99	.02	.51
HDL‐c^e^	91	0.91 (0.84‐0.98)	.02	0.89 (0.79‐0.99)	.04	0.86 (0.76‐0.98)	.02	.01	.33
LDL‐c^f^	173	1.05 (0.97‐1.13)	.22	1.06 (0.93‐1.20)	.36	1.08 (0.97‐1.21)	.17	.39	.45
Obesity traits									
BMI	432	1.50 (1.38‐1.63)	<.001	1.47 (1.25‐1.72)	<.001	1.47 (1.17‐1.85)	<.001	.21	.85
Waist circumference	357	1.70 (1.53‐1.89)	<.001	1.64 (1.36‐1.98)	<.001	1.52 (1.12‐2.05)	.008	.32	.43
Whole body fat mass	412	1.50 (1.37‐1.64)	<.001	1.51 (1.31‐1.75)	<.001	1.50 (1.16‐1.93)	.002	.05	.99
Trunk fat mass	399	1.48 (1.36‐1.62)	<.001	1.51 (1.31‐1.75)	<.001	1.02 (1.31‐1.68)	.03	.08	.29
Arm fat mass (left)	402	1.57 (1.43‐1.71)	<.001	1.52 (1.30‐1.77)	<.001	1.57 (1.22‐2.00)	<.001	.14	.99
Leg fat mass (left)	397	1.69 (1.51-1.90)	<.001	1.65 (1.37-1.99)	<.001	1.34 (1.83-2.53)	<.001	.11	.59
Metabolic syndrome									
T2DM^g^	148	1.03 (0.99‐1.07)	.16	1.05 (0.98‐1.11)	.14	1.07 (0.99‐1.15)	.11	.11	.31
Hypertension	55	1.00 (0.94‐1.06)	.98	0.97 (0.90‐1.06)	.56	0.86 (0.70‐1.05)	.15	.52	.13
Cardiovascular diseases	9	1.09 (0.91‐1.32)	.35	0.96 (0.75‐1.22)	.75	0.52 (0.21‐1.32)	.21	.66	.16
Ischemic stroke	8	1.02 (0.88‐1.18)	.78	0.99 (0.82‐1.19)	.90	1.10 (0.37‐3.24)	.87	.81	.90
Percent liver fat	10	1.02 (0.93‐1.11)	.66	1.02 (0.91‐1.13)	.76	0.96 (0.84‐1.10)	.59	.56	.30

a SNP: single-nucleotide polymorphism.

b IVW: inverse variance weighted.

c OR: odds ratio.

d WM: weighted median.

e HDL‐c: high‐density lipoprotein cholesterol.

f LDL‐c: low-density lipoprotein cholesterol.

g T2DM: type 2 diabetes mellitus.

### Educational and Psychological Factors

Considering the investigation of educational and psychological factors, we found that genetically predicted higher overall health rating (OR 2.19, 95% CI 1.61‐2.98; *P*<.001) was associated with an increased risk of sepsis (Table 2). Conversely, genetically predicted higher education attainment (individuals with a college or university degree; OR 0.52, 95% CI 0.40‐0.67; *P*<.001) was associated with decreased risk of sepsis. Notably, no significant causal association was found between genetically driven unhealthy emotions and sepsis (*P*=.40). We did not find statistically significant results when investigating reverse causality validation (*P*>.05).

### Metabolic Factors

We found an increased risk of sepsis associated with genetically predicted obesity. All fat mass–related traits were significantly associated with an increased risk of sepsis. The odds of developing sepsis increased with every 1‐SD increment in the BMI (OR 1.50, 95% CI 1.38‐1.63; *P*<.001), waist circumference (OR 1.70, 95% CI 1.53‐1.89; *P*<.001), whole body fat mass (OR 1.50, 95% CI 1.37‐1.64; *P*<.001), trunk fat mass (OR 1.48, 95% CI 1.36‐1.62; *P*<.001), arm fat mass (left; OR 1.57, 95% CI 1.43‐1.71; *P*<.001), and leg fat mass (left; OR 1.69, 95% CI 1.51‐1.90; *P*<.001; Table 2). However, high-density lipoprotein cholesterol (HDL-C; OR 0.91, 95% CI 0.84‐0.98; *P*=.02) exhibited a protective effect against sepsis. In contrast, no significant causal association was observed between genetically predicted triglycerides, total cholesterol, low-density lipoprotein cholesterol, type 2 diabetes mellitus, hypertension, cardiovascular diseases, ischemic stroke, percent liver fat, and sepsis (all *P*>.05).

### Reverse MR Analysis

To validate the reliability of the exposure-outcome causal direction, we performed reverse MR analysis by swapping the roles of the original exposure and outcome variables, and IVW analysis tests were conducted using the same set of SNPs. We did not find statistically significant results when investigating reverse causality validation (*P*>.05).

### Multivariable MR Analysis

**Figure 2.**
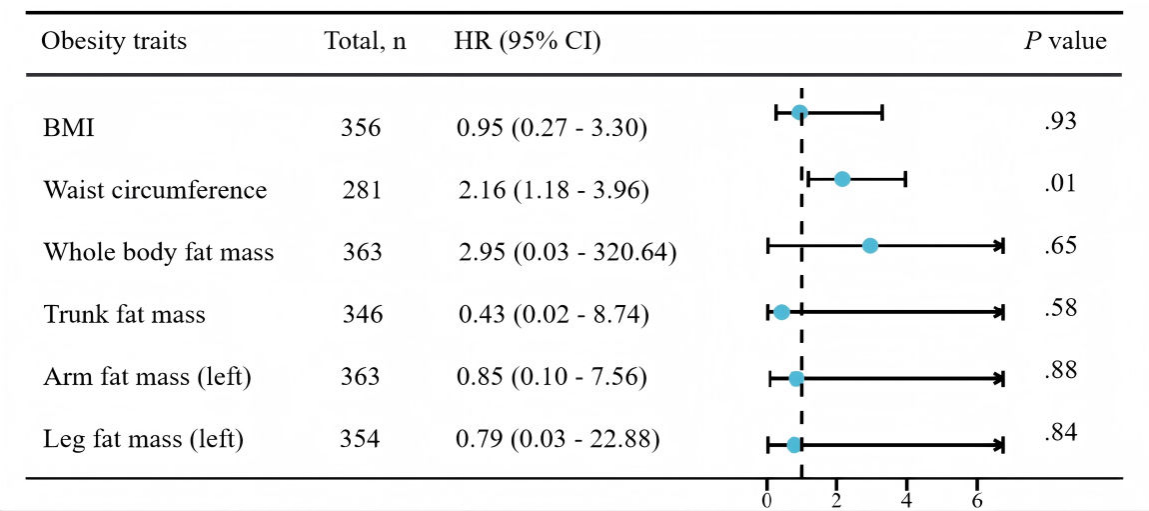
The association between obesity traits (BMI, waist circumference, whole body fat mass, trunk fat mass, arm fat mass, and leg fat mass) and sepsis by multivariable Mendelian randomization. Odds ratios (ORs) represent the associations with sepsis.

Considering the mutual influence of various obesity indicators, we conducted a multivariate MR analysis of BMI, waist circumference, whole body fat mass, trunk fat mass, arm fat mass (left), and leg fat mass (left). The results revealed that the waist circumference (OR 2.16, 95% CI 1.18‐3.96; *P*=.01) was an independent risk factor of sepsis (Figure 2).

### Heterogeneity and Pleiotropy Test

Although some results displayed heterogeneity based on the Cochran *Q* test (*P*>.05), the use of random-effects IVW as the primary outcome rendered this heterogeneity acceptable and did not undermine the validity of the MR estimates in this study (Table 2). In addition, the results were subjected to pleiotropy testing, demonstrating that those with significance did not exhibit pleiotropy. Therefore, assuming the absence of horizontal pleiotropy in this study is reasonable.

### Sensitivity Analysis

We conducted a sensitivity analysis using the MR-Egger intercept tests and the leave-one-out method. MR-Egger intercept tests showed no evidence of pleiotropy (Table 2). Using leave-one-out analysis, it is evident that nearly all lines align on one side of the y-axis, with minimal deviation beyond the axis. This consistency indicated the robustness of the results. The stable results shown in the MR visualization further corroborate the reliability of our study findings.

## Discussion

**Figure 3.**
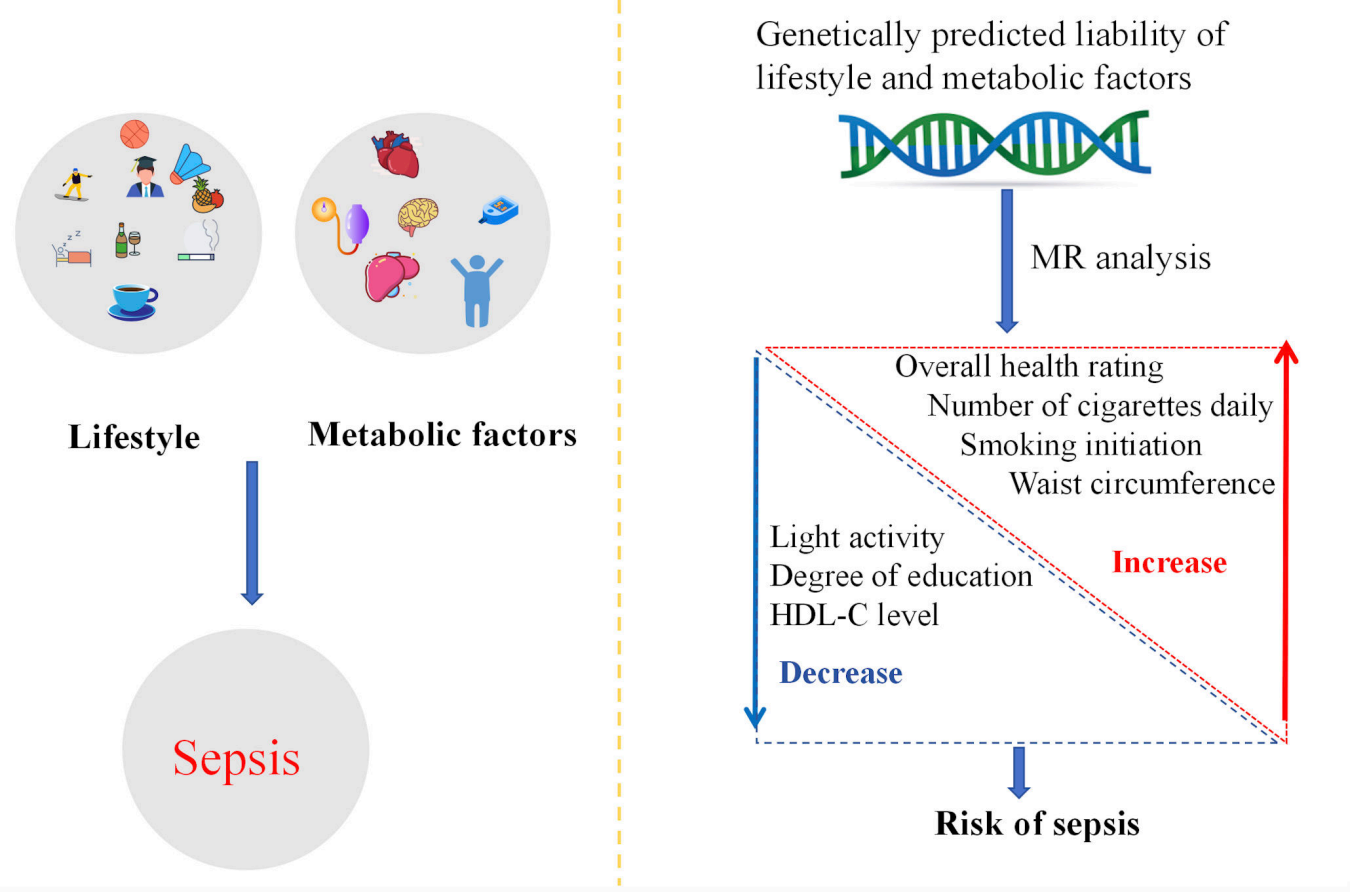
Association of modifiable lifestyle and metabolic factors with the risk of developing sepsis. HDL-C: high-density lipoprotein cholesterol; MR: Mendelian randomization.

In this bidirectional MR study, we investigated the association of lifestyle, educational and psychological, and metabolic factors with the risk of sepsis. Genetic variants such as smoking initiation, smoking frequency, and waist circumference were associated with an increased risk of sepsis, whereas light physical activity, higher education, and high levels of HDL-c were causally associated with a lower risk of sepsis (Figure 3).

Genetic variants such as smoking initiation, smoking frequency, and waist circumference were associated with an increased risk of sepsis, whereas light physical activity, higher education, and high levels of HDL-c were causally associated with a lower risk of sepsis.

Owing to the modifiable nature of lifestyle, the impact of lifestyle on diseases with high mortality, such as sepsis, has received increasing attention. Numerous observational studies have described an association between smoking, alcohol consumption, and the risk of various infectious diseases [22,30-35]. However, establishing a causal link between lifestyle and sepsis remains challenging. This study aimed to explore the potential causal relationships between lifestyle factors and sepsis. The results showed that smoking initiation and a high frequency of smoking were associated with an increased risk of sepsis. Previous immunologic experiments reported that smoking has been associated with impaired phagocytic function and cytokine expression of T lymphocytes and macrophages in the respiratory tract or peripheral blood [36-38]. These studies collectively underscore the detrimental impact of smoking on the immune system. These findings provide a better understanding of the role of smoking in patients with sepsis. Therefore, healthy lifestyle modification is a preventive measure against sepsis; individuals should be advised to reduce smoking to reduce the risk of developing sepsis.

Physical exercise is a planned, purposeful action to maintain a healthy lifestyle and improve physical fitness (World Health Organization, 2010) [39]. Furthermore, the evidence suggests that physical exercise affects multiple organ systems under various conditions [40]. It has been found that inadequate exercise is associated with a doubled risk of sepsis-related mortality [40]. Given that the incidence of sepsis is increasing, these results suggest that exercising could be immediately effective in reducing disease incidence. Currently, no MR analyses of the association between physical activity and sepsis are available. Our analysis confirmed that light physical activity is associated with a lower risk of sepsis.

The socioeconomic status affects health through environmental exposure, health behaviors, and lifestyle and has been shown to be positively related to health [10,41]. Education stands as the most potent indicator of socioeconomic status, exerting influence over lifestyle choices and access to health resources [42]. Observational studies suggest an inverse association between educational attainment and sepsis risk. Wang et al [43] reported significantly higher sepsis risk among individuals with lower education (hazard ratio 1.88, 95% CI 1.54‐2.29), though without controlling for comorbidities and other confounders. Another cohort study adjusting for socioeconomic factors still demonstrated a higher risk of sepsis-related intensive care unit admission in moderately educated versus highly educated individuals [44]. It should be noted that these observational findings may be influenced by residual confounding and do not establish causality. This study found a negative correlation between genetically predicted educational levels and sepsis risk. Higher education may reduce sepsis risk through multiple pathways, including enhanced health literacy (eg, earlier recognition of infections), greater access to health care resources, and adoption of healthier behaviors (eg, smoking cessation and improved diet). These mechanisms could collectively mitigate exposure to risk factors and improve timely medical intervention.

In recent years, an increasing number of studies have focused on the impact of metabolic factors such as blood glucose, blood lipids, and obesity on various infectious diseases [45-49]. Previous studies have reported that high HDL-c levels are significantly associated with reduced sepsis risk and other sepsis-related outcomes [50,51]. Extremely low serum HDL-c levels (≤20 mg/dL) are associated with an increased risk of death, sepsis, and malignancy [52]. However, observational clinical and epidemiologic studies have potential problems such as confounding, reverse causation, and unaccounted comorbidities. An MR study found that lower HDL-c levels were significantly associated with an increased risk of sepsis and related outcomes in infected patients [53]. This is consistent with our findings. Our study found a genetic relationship between high HDL-c levels and low risk of sepsis. HDL-c might exert protective effects against sepsis via its anti-inflammatory and antioxidant properties, as well as its role in modulating endothelial function and neutralizing bacterial endotoxins [54-56].

In observational studies on the general population, a higher BMI has been associated with an increased incidence of mortality from bloodstream infections and sepsis [57-59]. This finding is confirmed in this study. We also found that BMI, arm fat mass, leg fat mass, whole body fat mass, waist circumference, and trunk fat mass were significantly associated with sepsis. However, using the multivariate factor analysis, only increased waist circumference was an independent risk factor for sepsis, which might be because other factors included the causes of increased waist circumference, suggesting that these factors might influence sepsis risk as a consequence of shared risk factor profiles.

This study had several limitations. First, the population limited to the European ancestry hampered the generalization of the findings to individuals of other ancestries, as genetic and environmental factors influencing sepsis risk may vary across ethnic groups (eg, differences in inflammatory responses or health care access). Further studies are required to assess the modifiable risk of sepsis in other races. Second, as with all MR studies, pleiotropy in an MR setting is challenging. Additionally, gene-environment interactions (eg, how lifestyle modifies genetic risk) and potential measurement errors in exposure variables were not fully addressed, which might have introduced bias. We performed various sensitivity analyses that made different assumptions regarding the underlying nature of pleiotropy. Most tests showed stable results. Future studies should incorporate multi-ancestry cohorts and explore gene-environment interplay to strengthen causal inference.

In conclusion, our combined MR analysis supports the causal roles of smoking, educational level, HDL-c level, physical condition, obesity, and physical activity in patients with sepsis. The findings of this study highlight actionable targets for sepsis prevention. For instance, smoking cessation programs, public health initiatives to promote physical activity, and interventions to improve HDL-c levels (eg, dietary modifications or pharmacotherapy) could be integrated into personalized preventive strategies for managing high-risk populations. Furthermore, educational campaigns targeting health literacy and early symptom recognition might reduce delays in seeking care, particularly among socioeconomically disadvantaged groups. Future research should prioritize replication in diverse populations, mechanistic studies to elucidate pathways (eg, HDL-c’s immunomodulatory effects), and translational interventions targeting high-risk subgroups.

## Supplementary material

10.2196/72244 Checklist 1 STROBE-MR checklist.

## Abbreviations

GWAS: genome-wide association study
HDL-C: high-density lipoprotein cholesterol
IVW: inverse variance weighted
MR: Mendelian randomization
OR: odds ratio
SNP: single-nucleotide polymorphism
STROBE-MR: Strengthening the Reporting of Observational Studies in Epidemiology using Mendelian Randomization
